# The calcineurin/NFAT pathway is activated in diagnostic breast cancer cases and is essential to survival and metastasis of mammary cancer cells

**DOI:** 10.1038/cddis.2015.14

**Published:** 2015-02-26

**Authors:** C Tran Quang, S Leboucher, D Passaro, L Fuhrmann, M Nourieh, A Vincent-Salomon, J Ghysdael

**Affiliations:** 1 U1005-UMR3306-, Institut Curie, Bat 110 Centre Universitaire, Orsay 91405, France; 2 Institut National de la Recherche Santé et de la Recherche Medicale, Orsay U1005, France; 3 Centre National de la Recherche Scientifique, Orsay UMR3306, France; 4 Centre de Recherche, Institut Curie, Paris 75005, France; 5 CNRS UMR144, Paris 75005, France; 6 Department of Biopathology, Institut Curie, Paris 75005, France; 7 INSERM U934, Paris 75005, France

## Abstract

Nuclear factor of activated T cells 1 (NFAT1) expression has been associated with increased migratory/invasive properties of mammary tumor-derived cell lines *in vitro*. It is unknown, however, if NFAT activation actually occurs in breast cancer cases and whether the calcineurin/NFAT pathway is important to mammary tumorigenesis. Using a cohort of 321 diagnostic cases of the major subgroup of breast cancer, we found Cn/NFAT pathway activated in ER^−^PR^−^HER2^−^ triple-negative breast cancer subtype, whereas its prevalence is less in other subgroups. Using a small hairpin RNA-based gene expression silencing approach in murine mammary tumor cell line (4T1), we show that not only NFAT1 but also NFAT2 and their upstream activator Cn are essential to the migratory and invasive properties of mammary tumor cells. We also demonstrate that Cn, NFAT1 and NFAT2 are essential to the tumorigenic and metastatic properties of these cells in mice, a phenotype which coincides with increased apoptosis *in vivo*. Finally, global gene expression analyses identified several NFAT-deregulated genes, many of them being previously associated with mammary tumorigenesis. In particular, we identified the gene encoding a disintegrin and metalloproteinase with thrombonspondin motifs 1, as being a potential direct target of NFAT1. Thus, our results show that the Cn/NFAT pathway is activated in diagnostic cases of breast cancers and is essential to the tumorigenic and metastatic potential of mammary tumor cell line. These results suggest that pharmacological inhibition of the Cn/NFAT pathway at different levels could be of therapeutical interest for breast cancer patients.

Breast cancer is the first cause of death by cancer of women in developed countries. Its progression is characterized by distinct steps, starting with hyperplasia, *in situ* ductal carcinoma and invasive carcinoma, which can evolve into a deadly metastatic disease. Breast cancer is a complex disease in which tumor maintenance and progression to invasiveness relies both on tumor cell-intrinsic genetic lesions in oncogenes and tumor suppressor genes and on a constant dialog between tumor cells and their microenvironment.^1^

The nuclear factor of activated T cells (NFAT) family of transcription factors is composed of four Ca2+-regulated members (NFAT1-4) and one member regulated in response to osmotic stress (NFAT5). First identified as regulators of IL2 gene transcription in activated T cell, NFAT factors have since been shown essential to the development of diverse tissues (for review, see references Macian^2^ and Muller and Rao^3^). Classically, in unstimulated T cells, NFAT1-4 proteins reside in the cytoplasm in an hyperphosphorylated form. Activation of cell surface receptors coupled to Ca2+ mobilization from intracellular stores and ensuing opening of calcium-release activated channels (CRAC) leads to the activation of Ca2+-dependent enzymes, in particular, the calcineurin (Cn) protein phosphatase. Once activated, Cn catalyzes NFAT dephosphorylation, leading to its nuclear translocation. In the nucleus, NFAT factors regulate gene transcription, often in cooperation with unrelated transcriptional regulators. Cessation of Cn activation leads to the sequential rephosphorylation of nuclear NFAT by specific kinases and its export to the cytoplasm.

The implication of NFAT in oncogenic processes is beginning to emerge. First, the expression of a constitutively nuclear mutant of NFAT2 in immortalized 3T3 L1 fibroblasts leads to their transformation, suggesting an intrinsic role for NFAT in cellular transformation.^4^ Second, deregulation of NFAT expression or nuclear accumulation has been observed in several pathologies such as pancreatic,^5, 6^ prostate^7^ and in lymphoid malignancies.^8, 9^ In T-cell acute lymphoblastic leukemia (T-ALL), Cn is critical to the propagating activity of leukemic cells and controls nuclear accumulation of NFAT.^9, 10^ In breast carcinoma-derived cell lines, an Akt-dependent pathway regulating NFAT1 proteolytic degradation and *in vitro* cell migration and invasion has been described.^11^ Yet, the involvement of Cn in NFAT1 activation in this context is not established.^12^ Most importantly, the relevance of the activation of the Cn/NFAT module to breast cancer biology *in vivo* remains to be determined. To address these questions, we investigated whether the Cn/NFAT pathway is activated in diagnostic cases of breast cancer, and found Cn/NFAT module to be frequently activated in ER^−^PR^−^HER2^−^ triple-negative molecular poor prognostic subgroup. Using the 4T1 triple-negative mammary cell line, we show that NFAT1 or NFAT2 silencing impair the migration and invasion properties of tumor cells and that both NFAT1 and NFAT2 act downstream of Cn. Transcriptomic analysis identified over 300 genes, which are significantly deregulated in silenced NFAT1 cells, many of them being implicated in mammary tumorigenesis. In particular, we report that expression of the protease A Disintegrin And Metalloproteinase with ThromboSpondin motifs 1 (ADAMTS1), which was previously shown to be essential to mammary tumor development and metastasis,^13, 14^ is likely a direct target of NFAT1.

## Results

### The Cn/NFAT pathway is frequently activated in the triple-negative breast cancer subgroup

**Figure 1:**
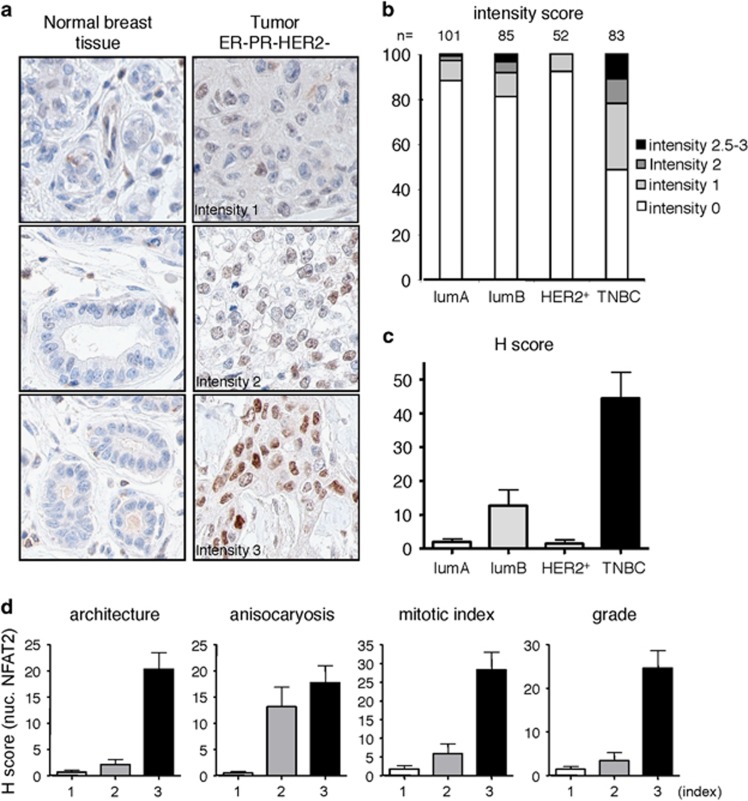
Nuclear localization of NFAT2 in hormone receptor-negative tumors. (**a**) Representative immunohistochemical staining of NFAT2 in sections of human breast tumors TMAs showing adjacent non neoplastic tissues (left panels) and ER^−^PR^−^HER2^−^ triple-negative tumors showing different intensity staining. (**b**) Intensity scoring of nuclear NFAT2 immunohistochemistry staining of tissue microarray (TMAs) of human breast tumors representative of the different molecular subtypes defined for their positivity to specific markers as follows: lumA: estrogen receptor (ER)>10%, progesterone receptor (PR)>20%, Ki67<14% LumB: ER>10%, PR<20%, Ki67>14% Her2+: ER<10%, PR<10%, HER2 2+ amplified; TNBC (triple negative): ER<10%, PR<10%, HER2 0/1 or 2+ non amplified according to ASCO guideline. The number of cases analyzed in the different subtypes is indicated at the top. (**c**) Nuclear NFAT2 H score (intensity × percentage of positively stained cells) in the different molecular subtype of breast cancer. (**d**) Nuclear NFAT2 H score according to the architecture, anisocaryosis, proliferative index and grade of the tumors

To investigate the activation status of Cn/NFAT module in breast cancer, we analyzed the expression and subcellular localization of NFAT in 321 primary breast tumors representative of the four main molecular subtypes of breast cancer (See Supplementary Table 1 for patients clinicopathological characteristics). As shown in Figures 1a and b, nuclear NFAT2 was detected in 42/83 of the ER^−^PR^−^HER2^−^ (TNBC; triple-negative breast cancer) tumors, whereas only a minority of the luminal A, luminal B and HER2+ tumors showed nuclear NFAT2 staining (12/101, 16/85 and 4/52, respectively). NFAT1 was also found nuclear in about half of the NFAT2-positive TNBC biopsies (see Supplementary Figure 1 for an example of NFAT1 nuclear staining). The H score of nuclear NFAT2, which takes into consideration the staining intensity in conjunction with the percentage of positively stained cells, was also found increased in ER^−^PR^−^HER2^−^ tumors as compared with the three other molecular subtypes (Figure 1c). These data show that nuclear accumulation of NFAT is observed in the most aggressive subtype of breast cancer. In line with this, the H score of nuclear NFAT2 was found higher in grade 3 tumors, which show the highest proliferative index and a highest disorganized architecture (Figure 1d). These results highlight the activation status of the Cn/NFAT pathway in the worst prognostic cases of breast cancer.

### CnB1, NFAT1 or NFAT2 silencing inhibits cell migration/invasion *in vitro*

**Figure 2:**
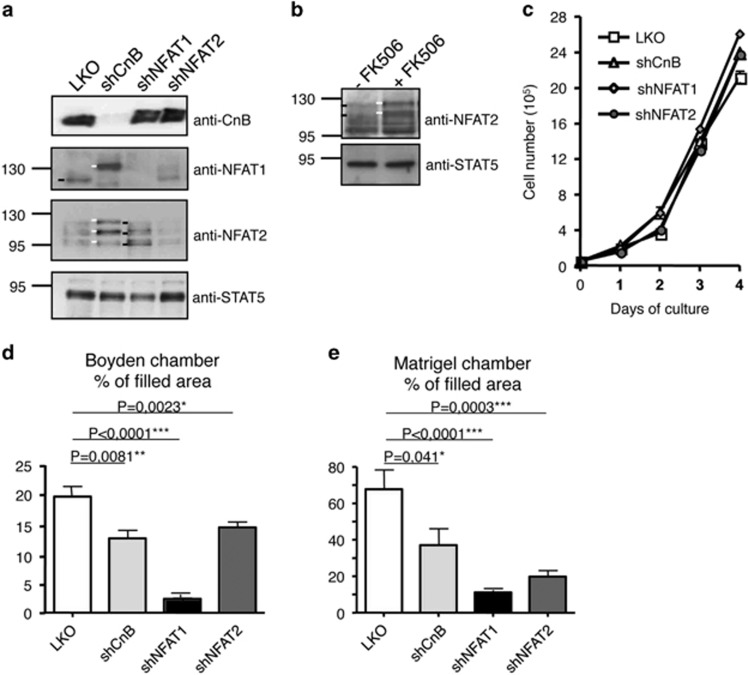
Silencing of CnB1, NFAT1 or NFAT2 in 4T1 cell impairs cells migration and invasion. (**a**) Extracts of shRNA-transduced and puromycin-resistant 4T1 cells, maintained for 1 week in culture, were immunoblotted for CnB1 (upper panel), NFAT1 (upper intermediate panel) or NFAT2 (lower intermediate panel) expression. The white traits point to the hyperphosphorylated isoforms of NFTA1 and 2, whereas black traits point to active, dephosphorylated isoforms of NFAT1 and 2. The blots were probed for STAT5 as normalizer. A representative blot of at least three independent experiment is shown. (**b**) As in (**a**) except using extract of 4T1 cells treated or not for 24 h with 50 nM FK506. (**c**) Growth of control and the indicated 4T1 cell derivatives maintained in culture in DMEM/F12+10% of FBS (*n*=3, data are represented as mean±S.E.M.). (**d**) 5 × 10^4^ of the indicated cells cultured in the absence of serum were seeded in the upper compartment of a Boyden chamber and assessed for their ability to migrate toward the lower compartment containing 10% FBS. After 16 h, the membranes were stained for the migratory cells and quantification of surface areas covered by migrating cells, performed using ImageJ. Data are the means of three independent experiments (*n*=3, data are represented as mean±S.E.M.). (**e**) As in (**d**) except 2 × 10^5^ cells were seeded for 24 h in modified Boyden Chamber loaded with Matrigel

Previous studies reported that NFAT1 is endowed with pro-migratory and pro-invasive capacities in breast cancer cell lines *in vitro* (see Introduction). We thus investigated whether this property is specific to NFAT1 and whether it depends on Cn activation. For this, the 4T1 mammary tumor cell line was stably transduced with lentiviruses expressing NFAT1-, NFAT2-, CnB1-specific small hairpin RNA (shRNA) or the pLKO as control. As shown in Figure 2a, this resulted in efficient and stable knockdown of the expression of the respective proteins. Of note, CnB1 knockdown resulted in NFAT1 and NFAT2 rephosphorylation, also observed in cells treated with a Cn inhibitor (Figure 2b). This indicates that Cn controls NFAT activation in mammary tumor cells. Inhibition of the Cn/NFAT signaling module in cells grown *in vitro* in 10% FBS did not affect their maintenance as no significant difference was observed between the expansion of control cells or cells silenced for either CnB1, NFAT1 or NFAT2 (Figure 2c). In contrast, under these conditions, CnB1, NFAT1 or NFAT2 silencing inhibited motility of 4T1 cells as assessed in Boyden chamber assays and by time lapse video microscopy (Figure 2d; Supplementary Figure 2 and 3) and impaired their ability to heal a wound (Supplementary Figure 3). Of note, the data of Supplementary Figure 3 used a distinct set of shRNAs for CnB1, NFAT1 or NFAT2, thus excluding nonspecific, off-target effects of the shRNAs used. Invasion was also analyzed using Boyden chambers with membranes precoated with a layer of Matrigel. CnB1, NFAT1 or NFAT2 knockdown also resulted in impaired invasion (Figure 2e). These results show that Cn and its major effectors NFAT1 and NFAT2 are important for breast cancer cells migration and invasion.

### The Cn/NFAT pathway is essential for mammary tumorigenesis

**Figure 3:**
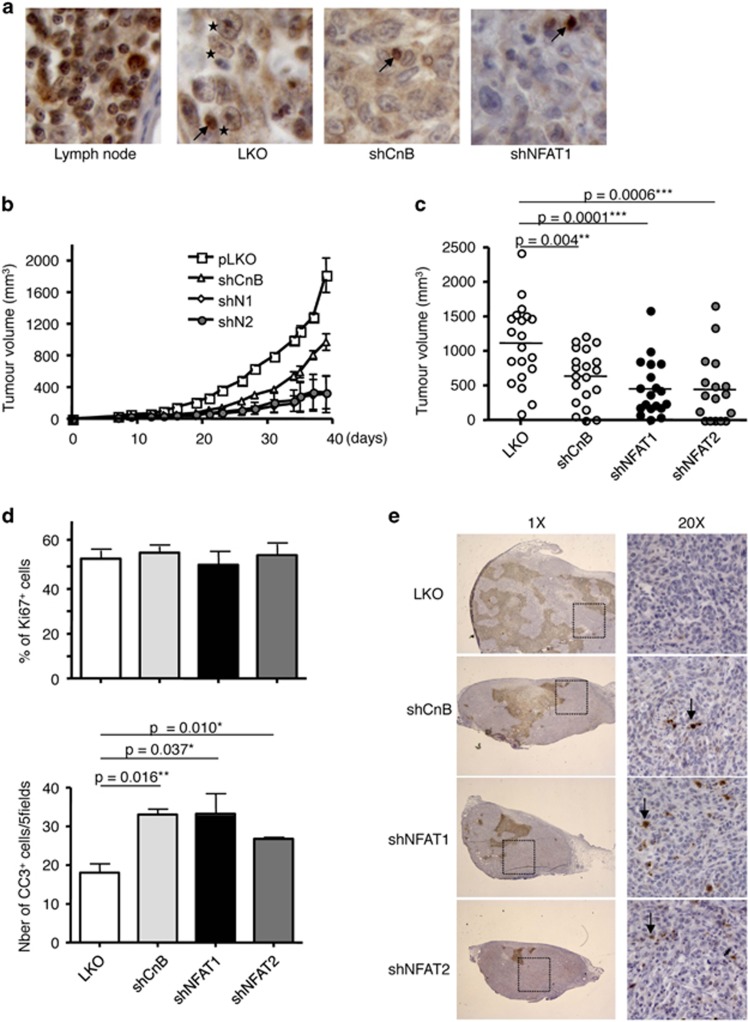
Knockdown expression of CnB, NFAT1 or NFAT2 impairs *in vivo* tumor growth. (**a**) Immunohistochemical analysis of NFAT1 expression and subcellular localization in control (LKO), CnB1-silenced (shCnB) and NFAT1-silenced (shNFAT1) 4T1 tumors. A lymph node section of control mice (left panel) has been used as positive control for NFAT1 expression and nuclear localization. Arrows point to NFAT1-expressing lymphoid infiltrating cells, whereas stars point to nuclear NFAT1 in mammary tumor cells. (**b**) 4T1 cells (5 × 10^5^; *n*=4 for each 4T1 cell derivatives) were inoculated in the inguinal mammary fat pad of syngeneic Balb/c mice. Tumor size was measured every 3 days with a caliper and the tumor volume calculated according to the equation *V*= (L × W^2^)/2. Data are represented as mean±S.E.M. (**c**) Tumor volume at the time of killing, 3–5 weeks after inoculation. Control pLKO tumors (white dots, *n*=21), shCnB1 tumors (light gray, *n*=21), shNFAT1 tumors (black dots, *n*=21), shNFAT2 tumors (gray dots, *n*=16). Data are represented as mean±S.E.M. (**d**) Quantification of the number of proliferative cells (Ki67+, upper panel) and apoptotic cells (CC3+, lower panel) in tumors (*n*=3) 15 days after inoculation. Numbers are the results of counting five consecutive fields at the × 20 magnification. Data are represented as mean±S.E.M. (**e**) Tumors excised 15 days after orthotopic injection were fixed, paraffin embedded and further analyzed for the expression of cleaved caspase 3 (CC3) by immunohistochemistry. The right panels represent a higher magnification of the insets shown in respective left panels. A representative picture of each tumor is shown. Arrows point to CC3-positive cells. Note the unspecific brown staining of necrotic areas observed at the × 1 magnification

To next determine whether the Cn/NFAT pathway is essential to tumorigenesis, the control and CnB, NFAT1 or NFAT2-silenced 4T1 cells were injected into the mammary gland of syngenic Balb/c mice and tumor growth was monitored every 3 days over a period of 3–6 weeks, time after which mice were killed. Immunohistochemical analysis of NFAT1 in 4T1 tumors revealed the activation of the Cn/NFAT pathway, as NFAT1 was detected in the nuclei (black stars) of control tumor cells, whereas it was exclusively found in the cytoplasm of shCnB1-silenced tumors and not detected in shNFAT1-silenced tumors (Figure 3a). As shown in Figures 2b and c, CnB1, NFAT1 or NFAT2 silencing resulted in a severe decrease in tumor size. Similar experiments realized with 168FARN cells, a less metastatic murine mammary tumor cell line, also show that NFAT1 or NFAT2 silencing resulted in decreased tumor size (data not shown). To understand the basis of the decreased size of CnB1-, NFAT1- and NFAT2-silenced tumors, tumors were analyzed 5 or 15 days after orthotopic engraftment for proliferation and apoptosis by Ki67 and cleaved caspase 3 immunohistochemical staining, respectively. No significant difference was observed in the proliferation/apoptosis rates, soon after cells injection (day 5, data not shown). Similarly, 2 weeks after engraftment, no difference was found in the proliferation rate of the different tumors (Figure 3d, upper panel). In contrast, a twofold increase in apoptotic cells was seen in CnB1-, NFAT1- and NFAT2-silenced tumors as compared with tumors transduced with the control vector (Figures 3d and e). These observations indicate that Cn, NFAT1 and NFAT2 are essential components of a survival pathway activated in mammary tumors *in vivo*.

### The Cn/NFAT pathway is essential for metastasis

**Figure 4:**
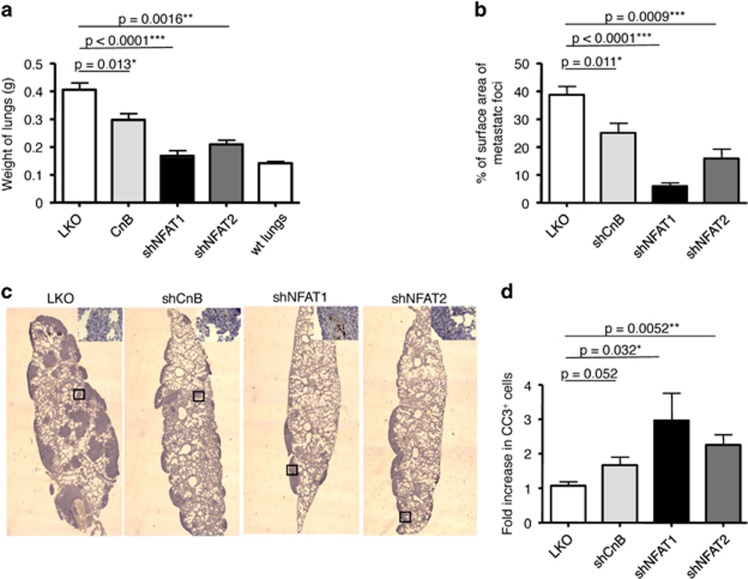
Inactivation of either Cn, NFAT1 or NFAT2 impairs the metastatic potential of 4T1 cells. (**a**) Weight of the lungs excised from killed mice, 2 weeks after intravenous injection of 5 × 10^5^ 4T1 cells. Note the significant decrease of the lung weight of mice injected with shCnB1-transduced cells (0.29±0.02 g, *n*=5), shNFAT1-transduced cells (0.16±0.02 g, *n*=5) or shNFAT2-transduced cells (0.21±0.01 g, *n*=3) cells *versus* control (LKO) 4T1 cells (0.40±0.02 g, *n*=5). Data are represented as mean±S.E.M. (**b**) Quantification of lung colonization after excision, fixation, paraffin embedding and H/E staining of lung sections of mice described in (**a**). Surface area of metastatic foci and surface of lungs were measured using ImageJ software. Data are represented as mean±S.E.M. (**c**) Representative pictures of lung sections as described in **b** after staining for cleaved caspase 3 and Hematoxilin/Eosin. Insets show a higher magnification of the underlined square. Note the brown CC3-positive cells specifically in shCnB, shNFAT1 and shNFAT2 metastatic foci. (**d**) Quantification of the number of apoptotic cells (CC3+) in metastatic foci of lungs, 15 days after intravenous injection of 4T1 cells. Data are plotted as fold increase compared with control and are the means of three independent tumors (*n*=3, data are represented as mean±S.E.M.)

In line with previous studies,^15^ metastatic cells were clearly detected in the lymph nodes, lungs and bones of mice carrying control tumors with an average size of 600 mm^3^ (see supplementary Table 2). In contrast, metastasis was profoundly inhibited in mice carrying similar sized CnB1, NFAT1 or NFAT2-silenced tumors, suggesting a function of Cn and NFAT in metastasis (Supplementary Table 2). To address more directly whether the Cn pathway is essential to the metastatic potential of breast cancer cells, 4T1 cells and its derivatives were directly injected in the tail vein of Balb/c mice. After 2 weeks, mice were killed and metastatic burden was analyzed. As shown in Figure 4a, mice injected with control 4T1 cells presented an increased weight of the lungs as compared with control mice (0.40±0.02 g *versus* 0.14±0.01 g), which correlated with the massive focal colonization of lungs by tumor cells (Figures 4b and c). In contrast, CnB1-, NFAT1-, NFAT2-silenced 4T1 cells were severely impaired in their ability to expand in lungs (Figure 4a), also shown in histological analysis (Figures 4b and c). Similarly to what was observed in primary tumors, an increase in apoptotic cells was seen in the metastatic nodules generated from NFAT1- and NFAT2-silenced 4T1 cells as compared with controls (Figures 4b and d). These results show that Cn and its NFAT1 and NFAT2 effectors are important both during the tumorigenic and metastatic processes, in part through their antiapoptotic properties.

### Characterization of the NFAT1-dependent transcriptome in 4T1 cells

**Figure 5:**
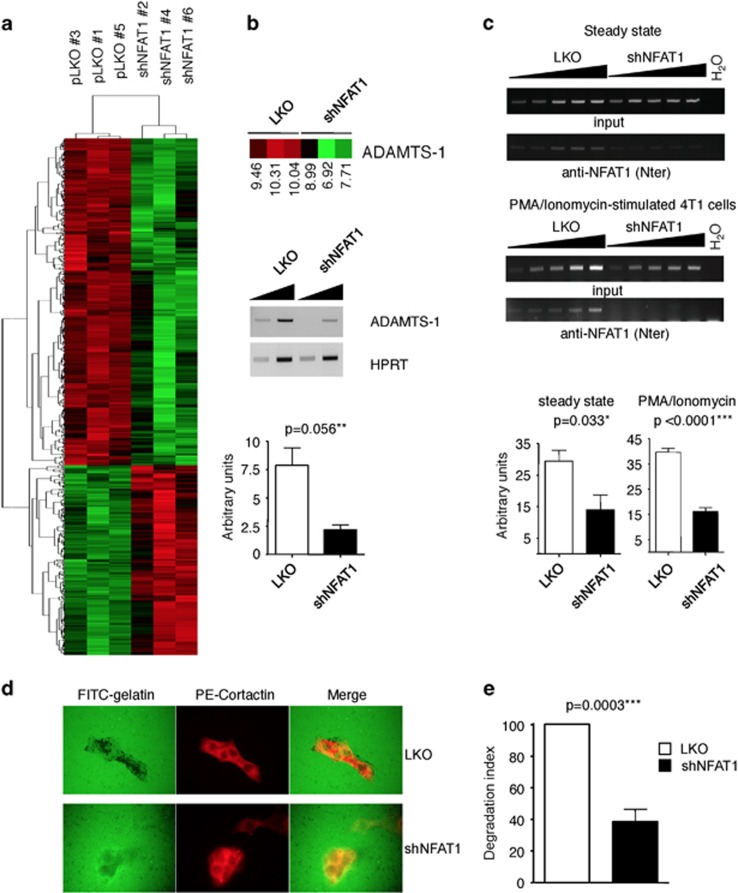
ADAMTS1 expression is downregulated in shNFAT1-4T1 cells. (**a**) Microarray analysis of three independent cultures of control (pLKO-transduced 4T1 cells) and shNFAT1-transduced 4T1 cells. Hierarchical clustering of the indicated 4T1 cells (top) in their control (pLKO) and NFAT1-silenced versions (shNFAT1) was performed as described in Materials and Methods. The heatmap representation highlights upregulated genes in red and downregulated genes in green. (**b**) ADAMTS1 is downregulated in shNFAT1-4T1 cells (Java Tree View extract of data shown in a, top panel). Semi-quantitative reverse transcription-PCR analysis of ADAMTS1 expression (middle panel) and quantification in lower panel are shown. HPRT was used to normalize the experiments. Quantification was made of three independent experiments (*n*=3, data are represented as mean±S.E.M.). (**c**) ChIP/PCR detection of ADAMTS1 promoter in chromatin obtained from 4T1 control (pLKO) and NFAT1-silenced (shNFAT1) 4T1 cells maintained either under steady state (upper panels) or stimulated with PMA/Ionomycin for 6 h to acutely activate NFAT (lower panels). Note the absence of detection of ADAMTS1 promoter co-immunoprecipitated with the anti-NFAT1 antibody in shNFAT1-4T1 cells. The histogram shows the quantification of the co-immunoprecipitated DNA in the different conditions used (*n*=5, data are represented as mean±S.E.M.). (**d**) NFAT1-silenced 4T1 cells cultured for 24 h on FITC-gelatin show decreased *in situ* protease activity compared with control 4T1 (pLKO) cells. (**e**) Quantification of experiments shown in **d**, as described in Materials and Methods. Data are represented as normalized degradation (degradation index), which was calculated as the area of degraded matrix per cell relative to control pLKO-4T1 cells (*n*=4, data are represented as mean±S.E.M.)

To investigate the molecular basis of NFAT pro-oncogenic function, the transcriptome of control and NFAT1-silenced cells were compared using pangenomic mouse GeneChip 430 2.0 arrays (Affymetrix). Unsupervised clustering analyses showed that three independently generated control 4T1 cell cultures clustered together and away from the cluster formed by three independent shNFAT1-4T1 cultures (Figure 5a). To identify NFAT-dependent biological pathways and functions, we conducted gene ontology and pathway analyses using the 325 genes that showed an at least twofold expression change, using ingenuity pathways analysis (IPA). Major functions expected to be essential to the tumorigenic potential of cells were significantly affected, including ‘cellular growth and proliferation', ‘cell death and survival' and ‘cell movement' (Table 1). As apoptosis was enhanced *in vivo* in NFAT1-silenced tumors, we analyzed in more details the set of genes ascribed to this phenotype. Table 2 shows the top 10 downregulated (−) and upregulated (+) genes in shNFAT1-4T1 cells as compared with control cells. Interestingly, 70% of these deregulated genes (14 out of 20) were previously reported as important to mammary tumorigenesis (indicated by the asterisks in Table 2), reinforcing the hypothesis of an essential role of the Cn/NFAT pathway in breast cancer. The deregulated expression of ADAMTS1, ROR1, FST, TXNIP and KLF2 between control and NFAT1-silenced 4T1 cells was confirmed by semi-quantitative RT-PCR (supplementary Figure 4). Interestingly, the same trend was observed for these genes in CnB1 and NFAT2-silenced cells, suggesting common molecular targets between Cn, NFAT1 and NFAT2 (supplementary Figure 4). We further focused on ADAMTS1, as this gene, which encodes a protease involved in mammary tumor growth and metastasis,^13, 14^ was found among the most downregulated genes in NFAT-silenced cells and also ascribed to cell movement in IPA analysis (data not shown). RT-PCR analysis independently confirmed the strong downregulation of ADAMTS1 expression in NFAT1 4T1 cells (Figure 5b). The promoter region of mouse ADAMTS1^16^ revealed the presence of a NFAT consensus DNA-binding site A/T GGAAA (A/N) (A/T/C) N. Chromatin immunoprecipitation using an anti-NFAT1 antibody was thus performed to investigate NFAT1 binding to ADAMTS1 promoter region. For this, control and shNFAT1-4T1 cells were either maintained under steady-state conditions or stimulated with PMA/Ionomycin to ensure full NFAT1 activation. As shown in Figure 5c, the promoter region of ADAMTS1 was recovered specifically from the NFAT1 immunoprecipitates of 4T1 cells, but not from the chromatin immunoprecipitates of NFAT1-silenced 4T1 cells. These results suggest that ADAMTS1 expression is under direct NFAT1 transcriptional control. As the pro-metastatic properties of ADAMTS1 have been shown to rely on its metallopeptidase activity^17^ and as ADAMTS1 is endowed with gelatinase activity,^18^ we next compared the ability of 4T1 and NFAT1-silenced 4T1 cells to degrade glutaraldehyde cross-linked gelatin. For this, cells were plated on FITC-gelatin and disappearance of fluorescence analyzed. As shown in Figure 5d and quantified in Figure 5e, control cells displayed large spots of digested gelatin, whereas NFAT1-silenced cells showed a decreased number of digested areas. This suggests that the inability of shNFAT1-4T1 cells to degrade the extracellular matrix, an essential process for tumor cells to invade and disseminate, is associated with the decreased NFAT1-dependent expression of ADAMTS1.

## Discussion

Although previously reported data showed a role for NFAT1 in breast cancer cell lines migration *in vitro*, it remained unclear whether the Cn/NFAT pathway is actually activated in breast cancer and whether this pathway is functionally relevant to breast cancer tumorigenesis. Our results provide the first evidence that the Cn/NFAT pathway is activated in diagnostic cases of breast cancer, with a preferential activation in ~50% in triple-negative subtypes. Targeting the Cn/NFAT pathway could thus be of therapeutic value in this aggressive subtype. Indeed, our results using triple-negative mammary tumor cells demonstrate the essential role of the Cn/NFAT pathway to both the tumorigenic and metastatic potential of these cells in mice. Global gene expression analyses highlighted several major cellular functions that are altered in 4T1 cells in which NFAT1 expression is decreased. In particular, we demonstrated that the gene encoding the protease ADAMTS1 is a direct target of NFAT1, and that its NFAT1-dependent regulation likely participates to the pro-invasive properties of 4T1 cells.

Previous reported results showed that Cn inactivation using pharmacological inhibitors did not result in NFAT inactivation, or impaired *in vitro* invasion.^12^ Thus, the role of Cn/NFAT pathway in tumor cell invasion remained unclear. Our findings showing that Cn inactivation through shRNA-mediated silencing of the expression of its CnB1 regulatory subunit results in NFAT rephosphorylation show that the Cn/NFAT pathway is active in 4T1 cells both *in vitro* and *in vivo* and that NFAT nuclear translocation relies on Cn activity. Similar results were found in human breast cancer cell lines (unpublished observation). Moreover, we demonstrate that Cn is essential *in vivo* to the tumorigenic and metastatic potential of 4T1 cells, thus favoring a pro-oncogenic role of Cn in disease progression in mammary carcinogenesis. Of note, the ORAI1-3 pore subunit of CRAC, which is an upstream regulator of NFAT activation in other cell types^19^ are involved in breast tumor cell migration and metastasis^20^ and are candidates as upstream activator for Cn. Previous data have linked NFAT1 to *in vitro* migration and invasion of mammary cancer cell lines.^11, 12^ Our loss-of-function studies show that NFAT1 or NFAT2 silencing is sufficient to alter tumorigenesis and metastasis. This may reflect a non-redundant function of these factors in breast carcinogenesis or reflect their mutual dependence in gene expression or protein function. For example, expression of the NFAT2/A isoform is under NFAT transcriptional control in T cells.^21^ Such a mechanism does not operate in 4T1 cells, as knockdown of NFAT1 failed to impinge on the expression of any of the NFAT2 isoforms (Figure 2a). Transcriptional regulation by NFAT factors in normal cell physiology most often involves their cooperation with unrelated transcriptional regulators,^3^ or their binding as homodimers to palindromic or close to palindromic response elements.^22, 23^ It is thus possible that critical NFAT-dependent genes in mammary tumor cells rely on the binding of NFAT1/NFAT2 heterodimers. Alternatively, NFAT1 and NFAT2 may have completely non-redundant functions in breast cancer and each regulate a set of specific genes, although our results showing that NFAT1 or NFAT2 silencing often results in similar gene deregulation does not favor this last hypothesis.

Epithelial-mesenchymal transition (EMT) is thought to be essential for tumor cells to disseminate from the primary tumor, intravasate and survive into the blood to finally extravasate and colonize secondary organs. Activation of Cn catalytic subunit has recently been described to be associated with the acquisition of mesenchymal properties of MCF7 cells on mitochondrial stress,^24^ whereas NFAT could participate to the TGFβ-induced EMT of MDA-MB-231 cells.^25^ We thus investigated whether NFAT1-silenced 4T1 cells presented a more epithelial phenotype compared with controls cells. However, the expression of neither E-cadherin, ESRP1 (epithelial markers) nor that of Snail, Twist, Fibronectin, Vimentin, Mmp9 (mesenchymal markers/inducers) were significantly deregulated in NFAT1-silenced cells (Supplementary Table 3). However, as the highly tumorigenic and metastatic potential of 4T1 may not rely on EMT,^26^ the requirement for Cn/NFAT for metastasis reported here likely relies on mechanisms other than EMT. For instance, apoptosis was clearly found enhanced in tumors and metastatic foci with impaired Cn/NFAT pathway, a phenotype that is associated with the deregulation of a number of genes implicated in apoptosis (Table 1 and Table 2). Further studies will be required to confirm and assess the importance of the deregulation of these genes in the Cn/NFAT-dependent phenotypes in mammary tumors.

We identified ADAMTS1 as a likely direct NFAT1 target involved in ECM degradation. ADAMTS1 encodes a protease highly upregulated in MDA-MB-231 subclones endowed with high metastatic potential.^27^ It has also been shown essential to mammary tumorigenesis in PyMT model of mammary tumor development^14^ and to metastasis in xenografted MDA-MB-231 cells.^13^ Interestingly, the decreased tumorigenesis observed in PyMT/ADAMTS1^−/−^ mice was characterized by increased apoptosis.^14^ It is thus tempting to speculate that decreased ADAMTS1 expression seen in NFAT1-silenced tumors contributes to their apoptotic phenotype. Yet, the relative importance of apoptosis *versus* invasion/migration in the Cn/NFAT-dependant tumorigenic process remains to be dissected. Our findings provide a new insight into the molecular mechanism underlying the regulation of ADAMTS1 in breast cancer through the Cn/NFAT pathway. As immunotherapy using an anti-ADAMTS1 antibody has been reported to be efficient against 4T1-induced tumorigenesis in mice,^28^ our study reinforces the idea of therapeutically targeting ADAMTS1 – and other NFAT targets – to prevent breast cancer metastasis.

Besides NFAT target genes or CRAC inhibitors, the Cn/NFAT pathway offers other possibilities for therapeutic intervention, for example, by direct targeting of Cn itself using Cn inhibitors such as cyclosporine A (CsA) or Tacrolimus (FK506). Recent studies have shown that treatment of MMTV-Neu transgenic mice with FK506 inhibited tumor growth, an effect that was ascribed to impaired NFAT4-dependent tumor angiogenesis.^29^ Our results suggest that part of the antitumorigenic effects of FK506 in this model also likely resulted from the inhibition of Cn in the tumor cells themselves. Interestingly, a significant reduction in the incidence of breast cancer was found in a retrospective clinical study of patients treated with CsA after renal and cardiac transplantation.^30^ This difference was ascribed to the impaired supportive function of immune stromal cells to tumor cells but may also reflect direct effects on tumor cells in response to Cn inhibition by CsA. However, besides induction of secondary cancers,^30^ CsA or FK506 are associated with a number of ill-characterized off-target effects that may limit their usefulness. The identification of upstream activators or downstream effectors of the CnB/NFAT pathway, as reported here, may represent a therapeutical alternative to these limitations.

## Materials and Methods

### Cell culture and knockdown

4T1 were maintained in cultured for >3 weeks in DMEM/F12 medium supplemented with 10% FBS. The two CnB1, NFAT1 and NFAT2-specific shRNA and the control vector (with unrelated shRNA LKO #71336) were from Open Biosystems/Thermo Scientific (GE Healthcare, Velizy, France) (LKO shCnB11 #487, GIPZ #131430; pLKO shNFAT1#12356, #12354; pLKO shNFAT2 #81925, #81926). Viral production was performed as previously described.^10^ 4T1 cells were selected with puromycin (1 *μ*g/ml) for 5 days.

### Migration/invasion assays

Matrigel invasion inserts and migration inserts (8-*μ*m pores) for 24-well tissue culture plates (BD Biosciences, Le Pond-de-Claix, France) were used as recommended by the manufacturer. 10% FBS was used as chemoattractant using 5 × 10^4^ cells (for migration assays) or 2 × 10^5^ cells (for invasion assays). Migrating cells were fixed with 20% ethanol and stained with 0.1% crystal violet (Sigma-Aldrich, St. Louis, MO, USA). For quantification of invasion/migration, the total area of stained cells was measured using the Threshold command of MetaMorph 6.2.6 (MDS Analytical Technologies, Sunnyvale, CA, USA) and divided by the total surface of the membrane (% of filled area).

### Fluorescent gelatin degradation assays

In total, 3 × 10^4^ 4T1 cells per 12-well plates were incubated for 16 h on FITC-conjugated cross-linked gelatin (Molecular Probes, Invitrogen, Cergy Pontoise, France) as previously described,^31^ fixed and processed for IF as previously described.^10^ Cells were imaged with the × 63 objective of a Leica DPRXA microscope equipped with a photometrics coolSNAP HQ camera and steered by Metamorph (Molecular Devices Corp., Sunnyvale, CA, USA). The total surface of the coverslip was screened for dark spots specific to matrix degradation that was divided by the total number of cells (cortactin positive) to define a degradation index. The degradation index of control LKO-4T1 cells was set as 100%.

### *In vivo* tumor models

BALB/c mice were injected orthotopically in the 4th mammary gland with 5 × 10^5^ 4T1 cells or its derivatives in 50 *μ*l of PBS. Tumor size was measured every 3 days using a caliper and diameter was calculated as (*W*^2^ × *L*)/2 where *W*=width and *L*=length. Mice were killed after 3–6 weeks. For intravenous injection, 5 × 10^5^ 4T1 cells were injected in the lateral tail vein and mice killed 15 days after injection. BalB/C mice were maintained under specific pathogen-free conditions in the animal facility of the Institut Curie. All experimental procedures were performed in accordance with the recommendations of the European Community (86/609/EEC) and the French National Committee (87/848) for the care and use of laboratory animals. All animal experiments were carried out under the supervision of JG, who was authorized by the director of the Veterinary Services of the Préfecture de l'Essonne, Evry, France (agreement number 91–7).

### Human samples and clinical and tissue microarray data

Samples of primary breast tumors surgically removed prior to any radiation, hormonal or chemotherapy treatment at Institut Curie from 2005 to 2006 have been analyzed. The clinical and pathological features of patients are summarized (Supplementary Table 1). Our series of diagnostic cases include 321 invasive ductal carcinoma representative of each molecular subtype of breast cancer defined as: luminal A (*n*=101): estrogen receptor (ER)>10%, progesterone receptor (PR)>20%, Ki67<14% Luminal B (*n*=85): ER>10%, PR<20%, Ki67>14% HER2^+^: ER<10%, PR<10%, HER2 2+ amplified (*n*=52); TNBC (triple negative, *n*=83): ER<10%, PR<10%, HER2 0/1 or 2+ non amplified according to ASCO guideline. TMA consisted of replicate of tumor core selected from whole-tumor tissue section of each tumor sample and a matched tissue core from adjacent non tumoral breast epithelium. Immunohistochemistry was performed using the Leica Bond-III automated immunostainer (Leica Microsystems, Nanterre, France) and the anti-NFAT2 antibody (Santa Cruz, Heidelberg, Germany; sc-7294) as previously described.^9^

Analyses were performed in accordance with the French bioethics law 2004–800 and the French National Institute of Cancer (INCa) Ethic Charter and after approval by the Institut Curie board and ethics committee, which waived the need for written informed consent from the participants. Women were informed of the research use of their tissues and did not declare any opposition for such search. Data were analyzed anonymously.

### Immunohistochemistry

Seven micrometer sections of paraffin-embedded organs were used for immuno-histological analysis using rabbit anti-NFAT1 (HPA008789, Sigma-Aldrich), rabbit anti-cleaved caspase 3 (mAb #9664; Cell Signaling Technologies, Leiden, the Netherlands), mouse anti Ki67 (KI67-MM1-CE-S Leica) and amplified using the Vectastain Elite ABC kit (Vector) and revealed with DAB Peroxydase Substrate Kit (Vector, Les Ulis, France). Analysis/counting was performed independently by two investigators.

### Affymetrix microarrays

Microarray analyses were performed using total RNA from three independent LKO-control and three independent shNFAT-1-4T1 cultures using the Murine Genome 430.2 array (Affymetrix, Santa Clara, CA, USA) according to manufacturer's instructions (http://www.microarrays.ustrasbg.fr). Raw feature data were normalized, and log_2_ intensity expression summary values for each probe set were calculated using robust multiarray average (package affy V1.4.32). Unsupervised hierarchical clustering analysis was performed using Java Tree view and Cluster programs.

### Immunoblotting

Antibodies used in this study are: mouse monoclonal antibodies, anti-NFAT1 4G6-G5 (Santa Cruz, sc-7294), anti-NFAT2 7A6 (Santa Cruz, sc-7294), anti-CnB1 (Sigma-Aldrich, CO581), anti-STAT5 C17 (Santa Cruz).

### Semi-quantitative RT-PCR analysis

Total RNA was isolated from 3 × 10^6^ cells using Trizol reagent (Invitrogen). In all, 0.5–1 *μ*g of RNA was reverse transcript using the kit ImProm II Reverse Transcription System (Promega, Charbonnieres, France) according to the manufacturer's instructions. PCR were performed using two increasing doses of cDNA as indicated by the increment sign in the figures. GOTaq DNA Polymerase (Promega) and the following protocols were used: 94 °C for 5 min, followed by 30 cycles of 94 °C for 1 min, 60 °C for 1 min and 72 °C for 1 min. The sequence of the primers used for amplification were as follows: ADAMTS1 forward: 5′-CAGTACCAGACCTTGTGCAGACCTT-3′ ADAMTS1 reverse: 5′-CACACCTCACTGCTTACTGGTTTGA-3′ HPRT forward: 5′-GCTGGTGAAAAGGACCTC-3′, HPRT reverse 5′-CACAGGACTAGACCTGC-3′ ROR1 forward 5′-CCCGATTTCCCAATTACATG-3′ ROR1 reverse 5′-AGATCGCTGGTTTCATTGGC-3′ FST forward 5′-ACCTGAGAAAGGCCACCT-3′ FST reverse 5′-AGCTTCCTTCATGGCACACT-3′ TXNIP forward 5′-CATGAGGCCTGGAAA-CAAAT-3′ TXNIP reverse 5′-ACTGGTGCCATTAGGTCAGG-3′ KLF2 forward 5′-GCCTGTGGGTTCGCTATAAA-3′ KLF2 reverse 5′-TTTCCCACTTGGGATACAGG-3′.

### Chromatin immunoprecipitation

Control and shNFAT1-4T1 cells were stimulated with PMA (25 ng/ml) and ionomycin (1 mg/ml) for 4 h, then fixed in 1% formaldehyde for 20 min at 37 °C and processed for chromatin immunoprecipitation as previously described (Lesault, 2002 #63) using 10 *μ*g of anti-NFAT1 antibody (Sigma-Aldrich). The presence of *ADAMTS1* promoter fragments in immunoprecipitated chromatin was detected by PCR using 5′-CGCTTTAGCCATGGTGCCCATGG-3′ and 5′-CGAAACAGCGCTGGGACCAGC-3′ as primers. PCR products were analyzed on agarose gel and quantified using ImageJ software. Unprecipitated DNA was used as input.

## Figures and Tables

**Table 1:** Top molecular pathways deregulated in shNFAT1 knocked down cells

**Molecular and cell function**	**Significance**
Cellular movement	3.00 × 10^−8^
Cell death and survival	1.94 × 10^−7^
Cellular development	2.88 × 10^−7^
Cellular growth and proliferation	2.88 × 10^−7^
Cellular function and maintenance	3.47 × 10^−5^

The 325 genes that showed an at least twofold expression change between control and shNFAT1 samples were submitted to pathway analysis using the Ingenuity Pathways Analysis (IPA).

**Table 2:** The top 10 downregulated and upregulated genes with a *P-*value <0.05 and ascribed to ‘apoptosis' are shown

**Gene's name**	**Log ratio**	**Relation to breast cancer**
WISP1*	−2.912	Induction of WISP1 correlates with invasive breast cancer oncogenesis and reduced type 1 cell-mediated cytotoxic immunity: a retrospective study (Klinke *et al.*,^32^)
SERPINE1*	−2.295	Plasma PAI-1 levels in breast cancer-relationship with clinical outcome (Ferroni *et a*l.,^33^)
FST*	−2.073	FST found upregulated in highly invasive MDS-MB-435 overexpressing alpha4beta6 integrins (Chen^34^)
ADAMTS1*	−2.067	The ADAMTS1 protease gene is required for mammary tumor growth and metastasis (Ricciardelli *et al.*,^14^)
NRP1*	−2.033	Neuropilin-1 is expressed by breast cancer stem-like cells and is linked to NFkB activation and tumor sphere formation (Glinka *et al.*,^35^)
NTS*	−2.024	The neurotensin receptor-1 pathway contributes to human ductal breast cancer progression (Dupouy *et al.*,^36^)
ROR1*	−1.970	ROR1 is expressed in human breast cancer and associated with enhanced tumor cell growth (Zhang *et al.*,^37^)
ADAM12*	−1.940	ADAM12 redistributes and activates MMP-14, resulting in gelatin degradation, reduced apoptosis and increased tumor growth (Albrechtsen *et al.*,^38^)
DAG1	−1.696	
ABCG2*	−1.510	Breast cancer resistance protein
IRF8*	1.616	Epigenetic inactivation of IRF8 in breast carcinoma (among other carcinoma lines; Lee *et al.*,^39^)
IFI16*	1.663	Differential regulation of estrogen receptor a expression in breast cancer cells by metastasis-associated protein 1 (Kang *et al.*,^40^)
HIST1H1C	1.799	
ITGA1	1.875	
NPTX1	1.890	
CXCR3*	2.004	C-X-C ligand 10 and C-X-C receptor 3 status can predict tamoxifen treatment response in breast cancer patients (Hilborn *et al.*,^41^)
KLF2*	2.217	Silencing of KLF2 by the histone methyltransferase EZH2 in human cancer (Taniguchi *et al.*,^42^)
EYA2	2.316	
TRIB2	2.414	
TXNIP*	2.680	Role of thioredoxin reductase 1 and thioredoxin interacting protein in prognosis of breast cancer (Cadenas *et al.*,^43^)

The asterisk indicates that the gene has been associated with breast cancer in the literature. Column 2 indicates the log ratio (ln2) measured in the Affymetrix screen. Column 3 lists one reference of the link between the indicated gene and breast cancer. NA, not applicable.

## Abbreviations

NFAT: nuclear factor of activated T cells
CnB1: calcineurin B1
T-ALL: T-cell acute lymphoblastic leukemia
ADAMTS1: A Disintegrin And Metalloproteinase with ThromboSpondin motifs 1
TNBC: triple-negative breast cancer
ER: estrogen receptor
PR: progesterone receptor
HER2: human epidermal growth factor receptor-2
