# Supplementary figures and images for: The calcineurin/NFAT pathway is activated in diagnostic breast cancer cases and is essential to survival and metastasis of mammary cancer cells
Source: Cell Death Dis. 2015 Feb 26;6(2):e1658–. doi: 10.1038/cddis.2015.14 (PMC4669815; doi:10.1038/cddis.2015.14)

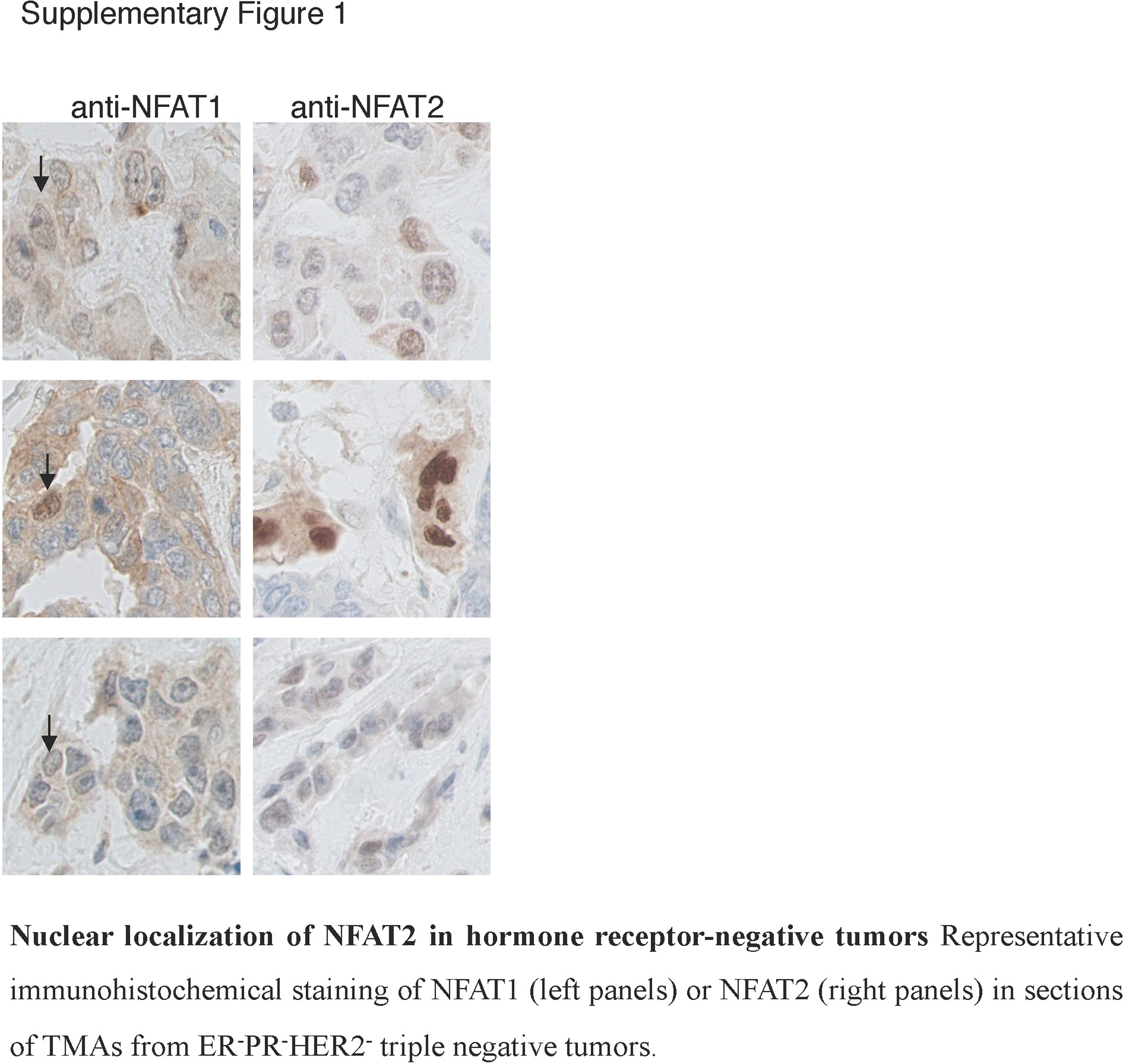

Supplement: Supplementary Figure 1 [file cddis201514x1.tif]

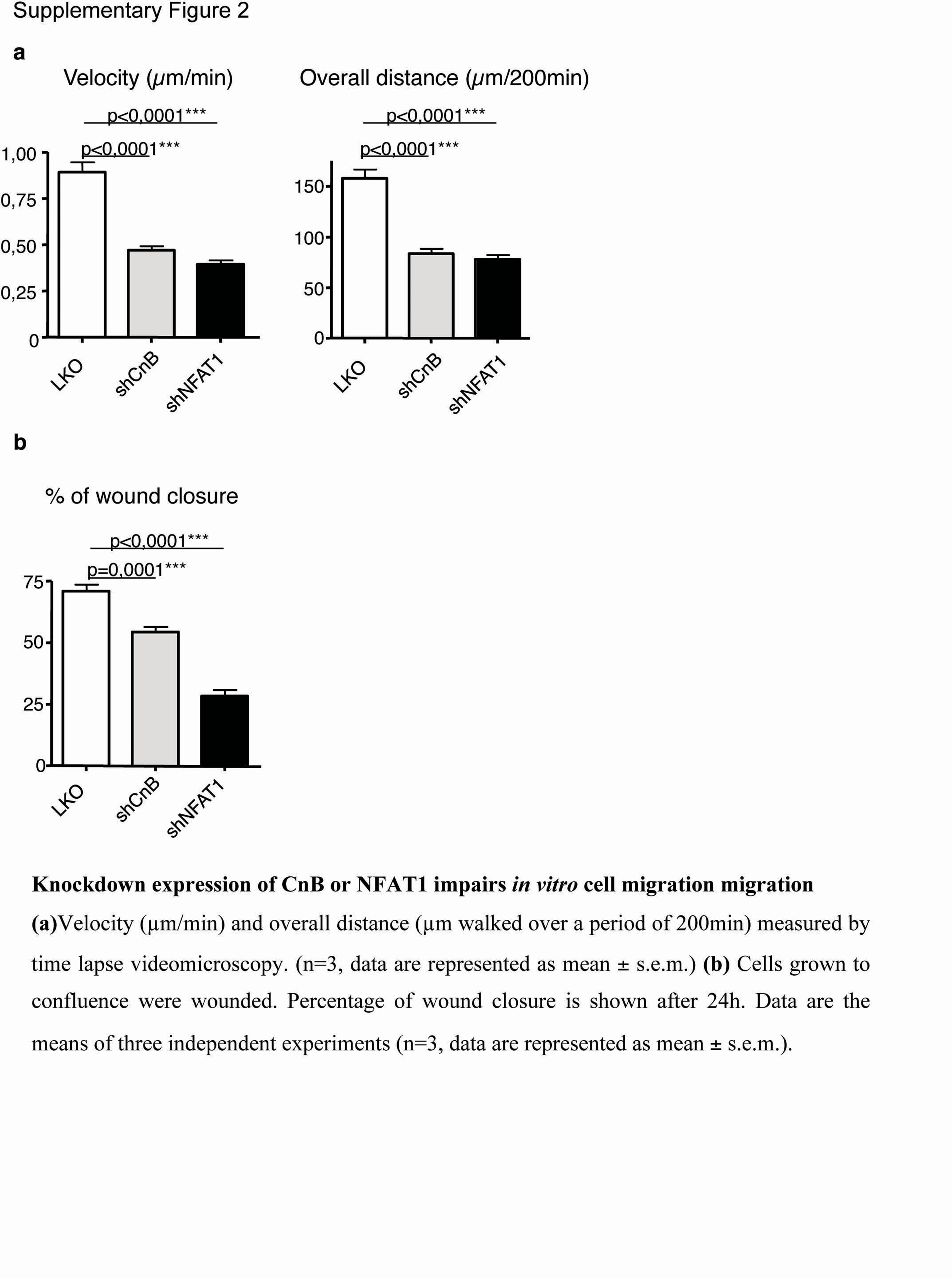

Supplement: Supplementary Figure 2 [file cddis201514x2.tif]

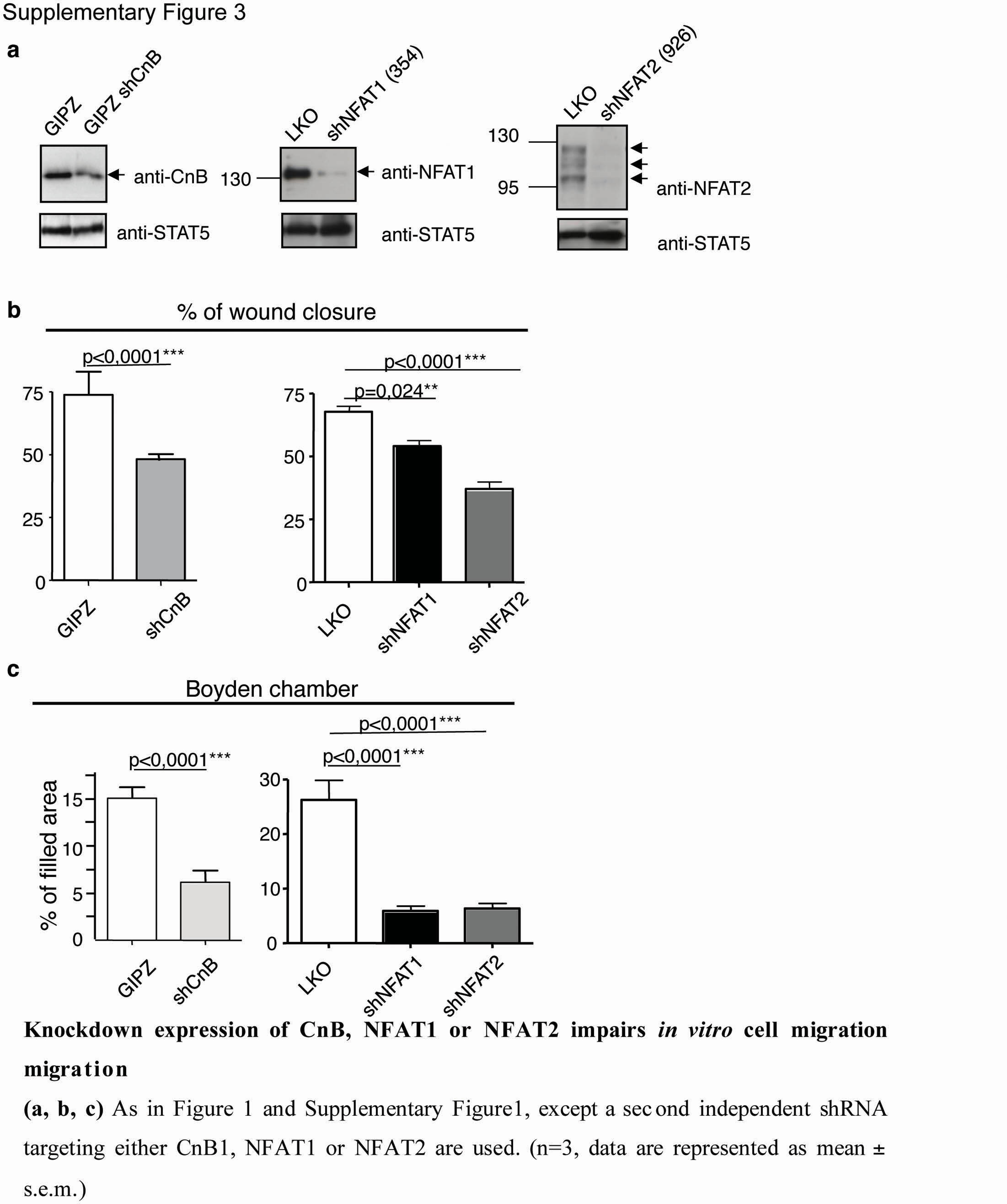

Supplement: Supplementary Figure 3 [file cddis201514x3.tif]

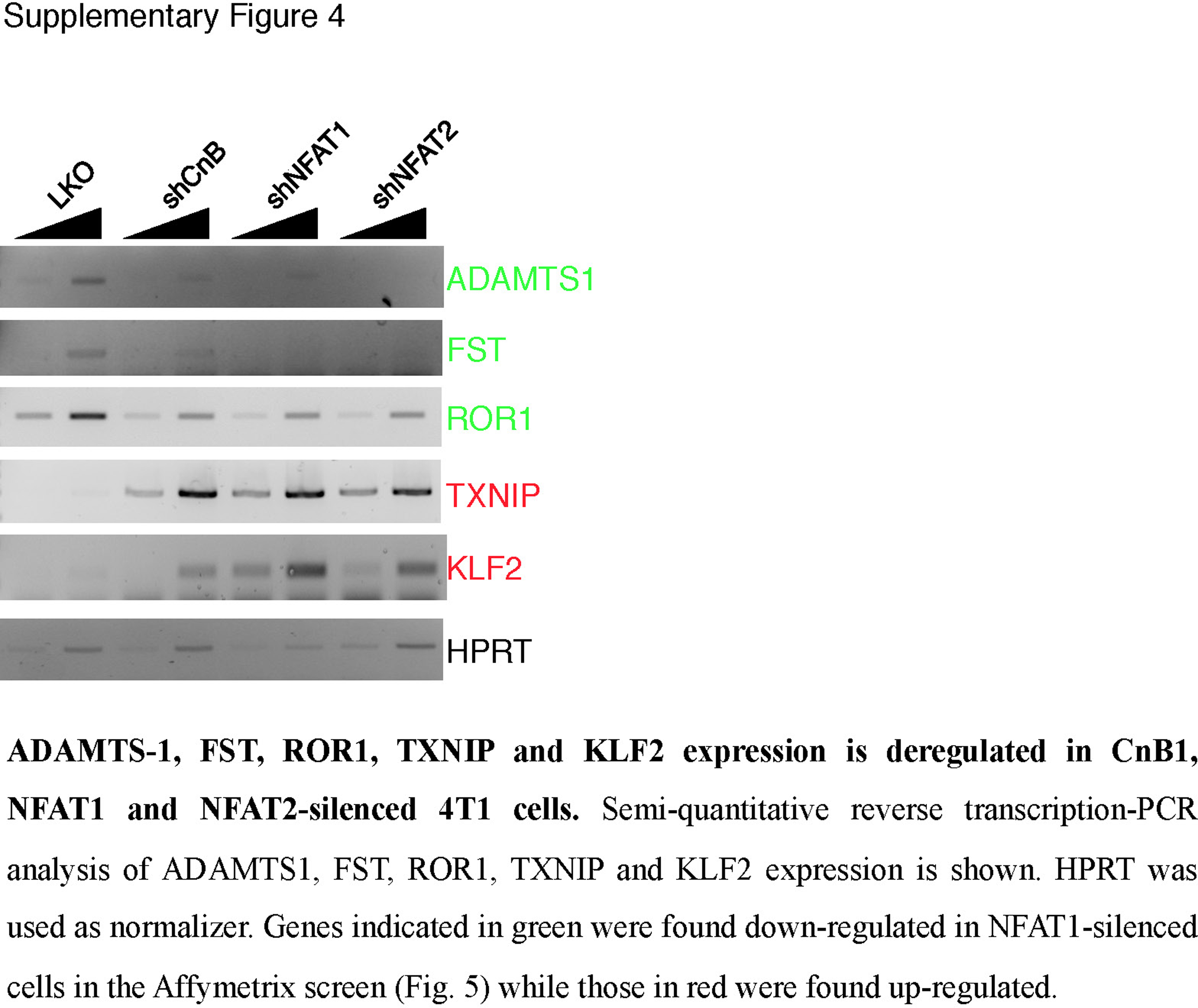

Supplement: Supplementary Figure 4 [file cddis201514x4.tif]
