# Supplementary material for: The calcineurin/NFAT pathway is activated in diagnostic breast cancer cases and is essential to survival and metastasis of mammary cancer cells
Source: Cell Death Dis. 2015 Feb 26;6(2):e1658–. doi: 10.1038/cddis.2015.14 (PMC4669815; doi:10.1038/cddis.2015.14)
Supplement: Supplementary Table 1 [file cddis201514x5.doc]

Supplementary Table 1

| **Clinicopathological characteristics of patients according to breast cancer phenotype** | | | |
| --- | --- | --- | --- |
|
|
|  | **FEATURES** | **Invasive carcinoma** | |
|  |  | **Total (n=321)** | ***(%)*** |
| **Age (years)** | |  |  |
|  | *<50* | 127 | *39,6* |
|  | *> 50* | 194 | *60,4* |
| **Menopausal status** | |  |  |
|  | *Premenopausal* | 116 | *36,1* |
|  | *Postmenopausal* | 188 | *58,6* |
|  | *Unknown* | 17 | *5,3* |
| **Histological grade (invasive tumors)** | |  |  |
|  | *I* | 53 | *16,5* |
|  | *II* | 97 | *30,2* |
|  | *III* | 171 | *53,3* |
| **Histological subtype** | |  |  |
|  | *Ductal carcinoma* | 315 | *98,2* |
|  | *Lobular carcinoma* | 3 | *0,9* |
|  | *Others* | 3 | *0,9* |
| **Tumour size (cm)** | |  |  |
|  | *T1 (<2)* | 211 | *65,7* |
|  | *T2 (2 - 5)* | 100 | *31,1* |
|  | *T3 (>5)* | 8 | *2,6* |
|  | *T4* | 2 | *0,6* |
| **N stage** | |  |  |
|  | *N0* | 181 | *56,4* |
|  | *N1* | 97 | *30,2* |
|  | *N2* | 31 | *9,7* |
|  | *N3* | 10 | *3,1* |
|  | *Unknown* | 2 | *0,6* |
| **ER** |  |  |  |
|  | *Positive* | 186 | *58* |
|  | *Negative* | 135 | *42* |
| **PR** |  |  |  |
|  | *Positive* | 168 | *52,3* |
|  | *Negative* | 153 | *47,7* |
| **HER2** | |  |  |
|  | *Positive* | 52 | *16,2* |
|  | *Negative* | 269 | *83,8* |
| **Ki67** | |  |  |
|  | *Positive (>20%)* | 235 | *73,2* |
|  | *Negative (<20%)* | 86 | *26,8* |
| **Molecular subtype** | |  |  |
|  | *TNBC* | 83 | *25,8* |
|  | *HER2* | 49 | *15,3* |
|  | *Luminal A* | 101 | *31,5* |
|  | *Luminal B* | 85 | *26,5* |
|  | *Luminal B / HER2* | 3 | *0,9* |
