# Supplementary material for: The calcineurin/NFAT pathway is activated in diagnostic breast cancer cases and is essential to survival and metastasis of mammary cancer cells
Source: Cell Death Dis. 2015 Feb 26;6(2):e1658–. doi: 10.1038/cddis.2015.14 (PMC4669815; doi:10.1038/cddis.2015.14)
Supplement: Supplementary Table 2 [file cddis201514x6.doc]

Supplementary Table 2

| Tumor | Lymph node | Lung | Bone |
| --- | --- | --- | --- |
| LKO | 2/3 | 3/3 | 1/3 |
| shCnB | 0/3 | 0/3 | 0/3 |
| shNFAT1 | 0/3 | 0/3 | 0/3 |
| shNFAT2 | 0/3 | 1/3 | 0/3 |

**Silencing of CnB1, NFAT1 or NFAT1 in 4T1 cell impairs their metastatic potential.** Mice carrying 4T1-derived tumors in which either CnB1, NFAT1 or NFAT2 expression had been knocked-down and of similar size (average 600 mm^3^) were sacrificed and analyzed for the presence of metastasis in lymph nodes, lungs and sternal bone. Numbers indicate the number of mice that present metastatic cells in the organ of interest as compared to the number of analyzed mice (n=3).
