# Supplementary material for: The calcineurin/NFAT pathway is activated in diagnostic breast cancer cases and is essential to survival and metastasis of mammary cancer cells
Source: Cell Death Dis. 2015 Feb 26;6(2):e1658–. doi: 10.1038/cddis.2015.14 (PMC4669815; doi:10.1038/cddis.2015.14)
Supplement: Supplementary Table 3 [file cddis201514x7.doc]

Supplementary Table 3


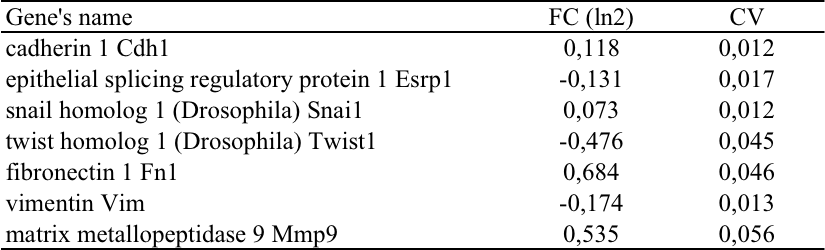


**Expression of EMT-associated genes in control (pLKO –tranduced 4T1 cells) and shNFAT1-transduced 4T1 cells.** The average fold change (FC) in expression and accompanying coefficient of variation (CV) of genes implicated in EMT between LKO (control) and NFAT1-silenced 4T1 cells were collected from the transcriptomic data shown in Figure 5.
